# Osteogenic Potential and Bone Matrix Maturity: Comparison of Demineralized Bone Matrix and P15 Polypeptide iFactor^®^ in an In Vitro Study

**DOI:** 10.3390/medicina61050914

**Published:** 2025-05-18

**Authors:** Anell Olivos-Meza, Monica Maribel Mata-Miranda, Marcelo Robles-Rodríguez, Gustavo Jesús Vázquez-Zapién, Melissa Guerrero-Ruiz, Carlos Landa-Solís

**Affiliations:** 1 Orthopedic Sports Medicine, Hospital Médica Sur, Mexico City 14050, Mexico; 2 Military School of Medicine, Centro Militar de Ciencias de la Salud, Secretaría de la Defensa Nacional, Mexico City 11200, Mexico; 3 Orthopedic Sports Medicine and Arthroscopy Department, Justo Sierra University, México City 07340, Mexico; 4 Tissue Engineering, Cell Therapy and Regenerative Medicine Unit, Instituto Nacional de Rehabilitación Luis Guillermo Ibarra Ibarra, Mexico City 14389, Mexico

**Keywords:** cartilage regeneration, scaffolds for cartilage repair, demineralized bone matrix, chondral tissue engineering

## Abstract

*Background and Objectives:* Demineralized bone matrix (DBM) is a widely used bone graft substitute due to its osteoconductive and osteoinductive properties. However, its efficacy varies due to differences in donor, processing, and storage conditions. Synthetic alternatives, such as iFactor^®^, combine non-organic bone mineral and a small peptide (P-15) to enhance the cellular attachment and osteogenesis. To compare the osteogenic potential and bone matrix maturity of iFactor^®^ and a commercial DBM scaffold through calcium nodule formation and Fourier transform infrared spectroscopy (FTIR) analysis. *Materials and Methods*: Human mesenchymal stem cells (hMSCs) were cultured and exposed to iFactor^®^ or DBM in paracrine culture conditions for 21 days. Calcium nodule formation was assessed using alizarin red staining and quantified spectrophotometrically. The FTIR analysis of hMSCs exposed to the scaffolds for three months evaluated the biomolecular composition and bone matrix maturity. *Results*: Calcium nodules formed in both groups but in smaller quantities than in the positive control (*p* < 0.05). The biomolecular components of the DBM were similar to healthy bone (*p* > 0.05) than those of the iFactor^®^ group (*p* < 0.005). A different rate of bone regeneration was observed through the formation of a greater number of calcium nodule aggregates identified in the extracellular matrix of mesenchymal stem cell cultures exposed to iFactor^®^ compared to those cultures enriched with DBM. *Conclusions*: Both experimental matrices demonstrated similar osteogenic potential at the 3-month follow-up. Although DBM has a closer biomolecular composition and carbonate substitution compared to healthy bone, iFactor^®^ showed faster matrix maturity expressed through the formation of a greater number of calcium nodule aggregates and higher hMSCs proliferation.

## 1. Introduction

Demineralized bone matrix (DBM) is commonly used as a bone graft substitute. Human demineralized bone matrix is classified as a transplant tissue in the United States, and it is processed and distributed by tissue banks [1,2,3,4]. DBM is an osteoconductive and osteoinductive commercial biomaterial extracted from human bone and retains a high quantity of proteins from native tissue with small amounts of calcium, inorganic phosphates, and some trace cell debris [5,6]. The osteoinductive potential of DBM varies due to different factors, including donor source, product storage conditions, decalcification process, washing procedures, and sterilization treatments [7,8]. The American Association of Tissue Banks (AATB) provides standards that DBM should contain less than 8% residual calcium, but most DBM formulations do not contain any residual minerals [9]. DBM products from the major bone graft suppliers have differing amounts of DBM content in their formulations; the remaining percentage is a carrier used to theoretically prevent the DBM powder from washing away from the defect site. Most DBMs mixed with a carrier have a DBM percentage between 17 and 40% by weight [9]. Depending on the form of the DBM, despite the described osteoinductive effect, its performance as a scaffold is inferior [2]. The effect is primarily determined by the content of remaining growth factors after processing, which may, unfortunately, be lowered due to processing, sterilization, storage, and donor characteristics [4,10]. Quality control of commercial DBM is crucial. Adding an excessive proportion of carriers (such as synthetic ceramic materials) [9] can significantly reduce the growth factors present in the DBM, directly impacting its effectiveness. DBM, in the field of orthopedic surgery, is used when it is necessary to repair defects in long bones that have lost the ability to self-regenerate, either due to trauma or tumor resection [11]. Additionally, due to its ease of molding, it is used with materials that promote osteoinduction, such as calcium sulfate (CaSO_4_, CS), thereby supporting the generation of new extracellular matrix at the implantation site [12]. Similarly, considering this property, it has been used in 3D printing models, mixed with ceramic materials for the printing of scaffolds used in spine surgery [13], and spinal repair and fusion [14].

The use of biomaterials in tissue engineering has been the focus of recent research for the regeneration of bone surrounding the site of an injury. The repair and reconstruction of bone defects cannot be achieved without bone graft substitutes. These substitutes must effectively replicate the natural bone environment. There has been a continuous effort to create the perfect bone graft substitute that replicates the three key characteristics of an autologous bone graft: osteogenesis, osteoinduction, and osteoconduction. However, no synthetically produced material has been found to exhibit all three properties. Nonetheless, numerous substances have proven to be clinically beneficial in promoting bone fusion [15].

Peptides, such as P-15 (15-amino acid peptide fragment), have been identified as a potential osteogenic molecule. The design of P-15 was based on the principle that short-chain amino acid peptides are essential for functional protein expression [16]. This osteogenic peptide would be advantageous compared to growth factors specialty in immunogenicity and the ability to be fixed at high density upon a substrate having a high degree of biological specificity [1]. P-15 is a synthetic 15-amino acid peptide identical to the cell-binding domain located in an alpha 1 chain of type I collagen, which is the primary component of the extracellular matrix of bone [17].

iFactor^®^ Biologic Bone Graft is a commercial P-15 combined with an anorganic bone mineral (ABM). Osteogenic precursor cells attach to the iFactor^®^ (P-15), which is intended to initiate the new bone formation cascade. ABM particles are a natural form of hydroxyapatite that actively triggers cellular attachment of osteogenic precursor cells, resulting in the production of natural amounts of bone morphogenic proteins and growth factors. Unlike growth factor products, iFactor^®^ only stimulates bone growth in the presence of bone-forming cells such as hMSCs. Research has demonstrated that P-15’s primary effect on bone tissue involves stimulating cell proliferation and enhancing cell adhesion. This occurs through the induction of growth factors including BMP-2, BMP-7, and TGF-β-1, collectively accelerating de novo bone formation at sites adjacent to P-15 application. iFactor^®^ does not require the presence of additional growth factors to stimulate the differentiation of bone progenitors. A key component of iFactor^®^ is P-15, which increases the production of BMP-2, BMP-7, and TGF-β1 in contact with bone progenitor cells. This has been demonstrated in the increased cell cohesion, proliferation, and differentiation into bone [9].

In clinical trials, iFactor^®^ achieves the union in lumbar fusion cases significantly earlier than rhBMP-2 and DBM, maintaining a favorable clinical and complication profile [18]. A cohort study was conducted in 2024 of 67 patients who underwent lumbar fusion treatment using ABM/P-15 as a bone graft, with a total of 100 lumbar levels. Fusion rates exceeding 200 Hounsfield units (HUs) were observed in 91.29% of patients as early as 3 months post-operation, measured by simple lumbar spine computed tomography [19]. Overall, no adverse events were reported for this product.

As previously described, DBM is an effective tool for bone induction [11,12,13,14]. However, since each batch of DBM is derived from different donors, it is impossible to ensure that there is no variability among its components. This variability arises from factors such as ethnicity, sex, age, or the nutritional status of the human donors [20,21,22,23]. This is the importance of synthetic molecules like iFactor^®^, which can offer a similar osteoinductive effect to DBM, due to iFactor^®^’s synthetic components that are always standardized, regardless of the place in the world they are used, thus yielding homogeneous and reproducible results. For this reason, this study aimed to evaluate and compare the cellular biocompatibility and osteogenic potential of two commercially available bone graft scaffolds, iFactor^®^ and DBM, using human mesenchymal stem cells (hMSCs).

## 2. Materials and Methods

### 2.1. Handling of Human Mesenchymal Stem Cells

Commercially available mesenchymal stem cell line derived from human bone marrow was used (hMSCs, cat. PT-2501, Lonza, Basel, Switzerland) to evaluate matrices with osteogenic potential. According to the information provided by the Loza team, the hMSCs were obtained through bone marrow donation via iliac crest puncture after the donor signed the informed consent form. The donor’s characteristics were as follows: 21 years old, male, with negative viral tests for HIV, HBV, HCV, a negative result for mycoplasma, and a viability of 94% at the time of cell freezing during the first passage. The immunophenotype showed that the cells were positive for CD105 (98%), CD166 (99%), CD44 (98%), CD90 (97%), and CD73 (93%), and negative for CD14 (0%), CD34 (0%), CD45 (0%), HLA-DR (1%), and CD19 (0%). Finally, the hMSCs passed an adipogenic differentiation test.

After receipt in the laboratory, the hMSCs were thawed and expanded in culture using the Mesenchymal Stem Cell Growth Medium Bullet Kit (cat. PT-3001, Lonza, Basel, Switzerland) until the required cell number for the experiments was obtained. To preserve the cells for future use, they were cryopreserved in 1 mL vials containing 1 × 10^6^ cells and stored in a cryopreservation tank (liquid nitrogen at −196 °C) until their use.

### 2.2. Evaluation of Calcium Nodules (Ca^2+^) Formation Capacity

**Figure 1:**
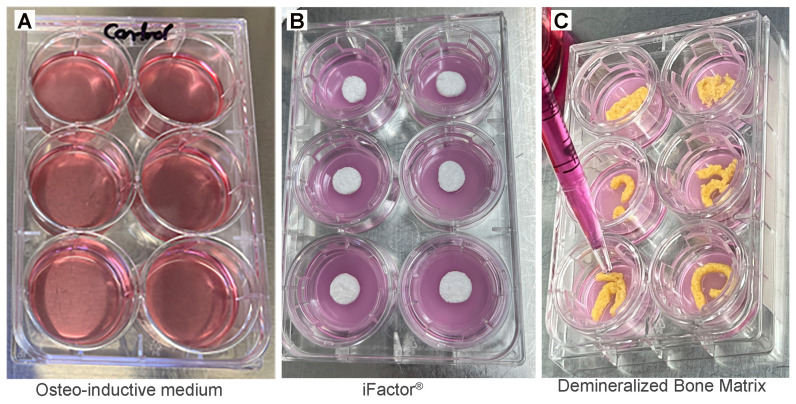
Macroscopic photographs of culture conditions to evaluate the calcium nodule formation potential (Ca^2+^) involved three experimental setups. (**A**) 1 × 10^5^ human mesenchymal stem cells (hMSCs) were directly seeded as a monolayer in a 6-well plate and stimulated with osteoinductive medium. (**B**) illustrates a co-culture condition in which the same number of hMSCs were seeded in a monolayer, with a transwell insert containing 100 mg of iFactor^®^ placed above the cells. Finally, (**C**) shows another co-culture setup, where 1 × 10^5^ hMSCs were seeded in a monolayer with a transwell insert containing 100 mg of demineralized bone matrix (DBM). All cultures were maintained and monitored for 21 days to assess the progression of osteogenic differentiation.

Given the ability of hMSCs undergoing osteodifferentiation to form calcium nodules in their extracellular matrix [24], alizarin red staining (cat. A5533, Sigma-Aldrich, San Luis, MO, USA), was used to assess the degree of mineralization in the extracellular matrix of hMSCs exposed to iFactor^®^ or biologic demineralized bone matrix (DBM) in paracrine culture. To achieve this, 1 × 10^5^ hMSCs were seeded per well in triplicate on 6-well plates with trans-well inserts (cat. 3428, Corning, Nueva York, NY, USA), along with the positive control using osteo-inductive medium (cat. 130-091-678. Miltenyi, Bergisch Gladbach, Germany). In the insert placed on top, 100 mg of iFactor^®^ or DBM was added, supplemented with DMEM medium containing 10% FBS and 1% antibiotic/antimycotic (All from Gibco, Waltham, MA, USA). The culture medium was changed every two days, and the culture was maintained for 21 days according to the manufacturer’s instructions for the positive control to induce osteo-differentiation of the hMSCs (see Figure 1).

After the incubation period, the supernatant was removed from the culture dishes, and the dishes were washed twice with PBS (Gibco, Waltham, MA, USA). The cells were then fixed with 4% paraformaldehyde (PFA, Sigma-Aldrich, San Luis, MO, USA) for 20 min, followed by an additional wash with running water. The cells were allowed to air dry, then stained with 2% alizarin red for 20 min in an orbital shaker at 40 rpm. The dye was removed, and the cells were washed with running water and left to dry. The formation of Ca^2+^ nodules was identified by small or large pleomorphic clusters ranging from 0.5 to 10 µm in red color (calcium deposits characteristic of osteogenic differentiation) under an inverted microscope with visible light. Subsequently, alizarin red was quantified by dissolving it in isopropanol for 20 min while shaking at 150 rpm. Finally, the absorbance of the dye was measured in a microplate reader at 415 nm (DTX 800, Beckman Coulter, Indianapolis, IN, USA). The absorbances were expressed as a percentage of cells, using the absorbance of the positive control group (or cells stimulated with osteo-inductive medium) as a reference for 100%.

### 2.3. Fourier Transform Infrared Micro-Spectroscopy Analysis (FTIR): 3-Month Follow-Up

**Figure 2:**
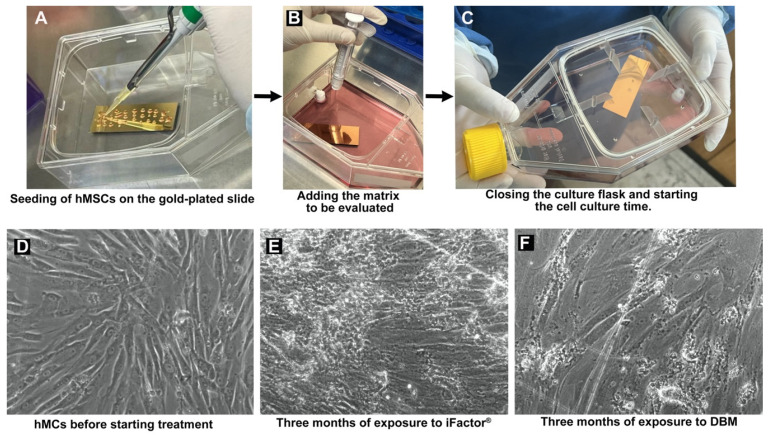
The process of seeding hMSCs onto the gold-plated slide and the microphotographs taken after three months of treatment with the evaluated matrices are shown. (**A**) The technique used to seed the cells onto the slide is illustrated by placing small drops (10 µL) until most of the slide area is covered; (**B**) after the cells were seeded and adhered (**D**), the matrix to be tested (iFactor^®^ or DBM) was added; (**C**) once the matrix was applied and the culture medium was added, the culture flask was sealed, and the 3-month incubation began; (**D**) microphotograph of hMSCs adhered to the slide, two days after seeding; (**E**) microphotograph of cells after 3 months of exposure to iFactor^®^, where the generation of aggregates resembling calcium nodules was macroscopically observed, as identified in the results obtained after 21 days of culture; and (**F**) microphotograph of hMSCs after 3 months of culture with DBM, where a lower proportion of aggregates in the extracellular matrix was observed.

For infrared analysis, 5 µm slices of healthy bone were first analyzed, followed by 1 × 10^6^ hMSCs seeded on gold-coated slides (100 nm gold layer thickness, Aldrich; Burlington, MA, USA), exposed to iFactor^®^ or DBM, and cultured in tissue culture flasks with reclosable lids (115 cm^2^ surface area; cat. TPP-90552, TPP, Trasadingen, Switzerland) containing DMEM medium with 10% FBS and 1% antibiotic/antimycotic for 3 months. The samples were analyzed using a 16× objective in an FTIR microscope (Jasco; IRT-5200, Tokyo, Japan), and the spectra were collected with an FTIR spectrophotometer (Jasco; 6600, Tokyo, Japan). For the procedure, the slides with the samples were focused and allowed to dry for 20 min. Once all moisture was removed, 140 scans were performed within a range of 4000–400 cm^−1^ at a resolution of 4 cm^−1^. FTIR absorbance spectra were obtained using Unscrambler X software (version 10.3). Jasco Spectra Manager software (version 2), was used to adjust the spectral range to 1800–800 cm^−1^ for the analysis of the biological fingerprint via FTIR spectrum acquisition. After three months of cultivating hMSCs under different experimental conditions, the cell culture process and morphology are shown in Figure 2.

### 2.4. Statistical Analysis

The results obtained from the quantification of alizarin red staining and FTIR analysis were analyzed using the Shapiro–Wilk normality test, which showed that the data followed a normal distribution. All variables mentioned above were analyzed using the Student’s *t*-test for paired samples, with the healthy or positive control compared to the cells exposed to iFactor^®^ or DBM, as appropriate. To assess the significance of all analyses, a *p*-value of 0.05 was used, and data were analyzed with SPSS software, version 17.0 (SPSS Inc., Chicago, IL, USA).

## 3. Results

### 3.1. Calcium Nodules (Ca^2+^) Formation Capacity

**Figure 3:**
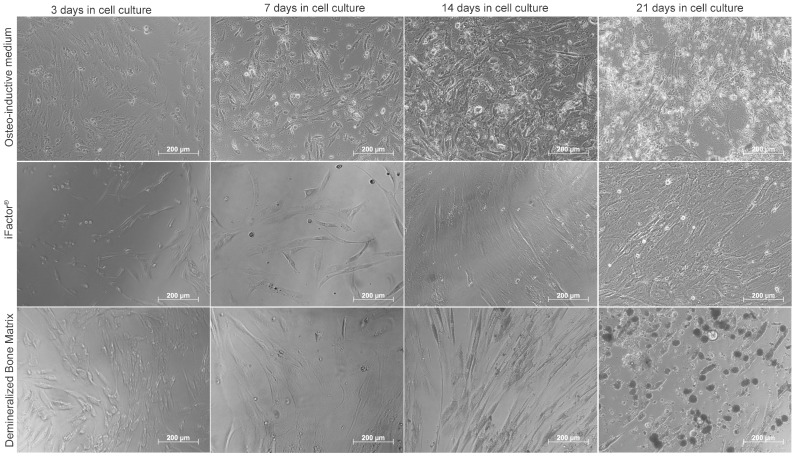
Microphotographs of the culture and stimulation process of hMSCs in monolayers at 21 days: For cells cultured with osteoinductive medium, a visible increase in cell number was observed up to day 14, followed by the appearance of whitish nodules (clusters of cells). In the case of the iFactor^®^-treated group, a decrease in cell number was evident between days 3 and 7, followed by a gradual increase from day 14 to day 21, at which point nodule formation was observed, although it was not comparable to that seen in the control group (osteogenic medium alone). Finally, in cultures exposed to demineralized bone matrix (DBM), cells maintained a similar morphology to the control group between days 3 and 7. A visible increase in cell number was noted by day 14, and by day 21, cells had clustered together to form nodules that appeared denser than those observed in both the control and iFactor^®^ groups.

After 21 days of culture, hMSCs exposed to the different experimental conditions underwent noticeable morphological changes throughout the treatment period (see Figure 3).

**Figure 4:**
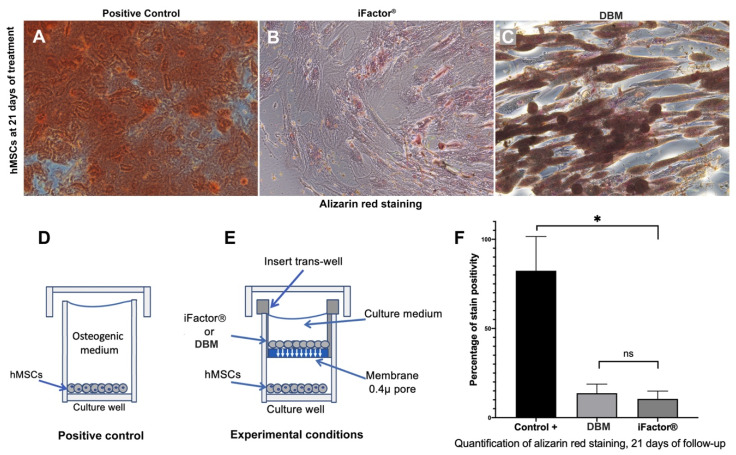
Evaluation of calcium nodule (Ca^2+^) formation capacity after 21 days of exposure to different experimental conditions using alizarin red staining. (**A**) hMSCs positive for alizarin red staining exposed to the osteo-inductive medium; (**B**) hMSCs exposed to iFactor^®^; (**C**) hMSCs exposed to DBM; (**D**) a schematic view of how the hMSCs were cultured with the osteo-inductive medium in a well of a cell culture plate; (**E**) a scheme showing how the hMSCs were treated with iFactor^®^ or DBM, using a trans-well insert placed on top of a culture well plate; (**F**) the graph of the analysis of staining intensity percentage, determined by optical density in a spectrophotometer. * Indicates a statistically significant difference (*p* < 0.05); ns = not significant.

For calcium nodules (Ca^2+^) formation capacity, the positive control showed extensive areas of the extracellular matrix positive for alizarin red staining (calcium nodules; Figure 4A). In the case of iFactor^®^, areas positive for calcium deposits were observed, but at a much lower intensity than in the control group (*p* < 0.05) (Figure 4B). On the other hand, DBM caused contraction of the extracellular matrix, which reduced the area positive for staining below the values of the positive control (*p* < 0.05) (Figure 4C). After quantifying the alizarin red staining, we found a statistically significant difference between the positive control and the two experimental groups (*p* = 0.003), both of which showed a lower percentage of positivity to the staining on average (iFactor^®^ = 10.53% ± 4.3 vs. DBM = 13.70% ± 5.09). However, although the DBM average was higher than that of iFactor^®^, the difference between both matrices did not reach statistical significance (*p* = 0.54; Figure 4F).

### 3.2. Fourier Transform Infrared Spectroscopy Analysis

**Figure 5:**
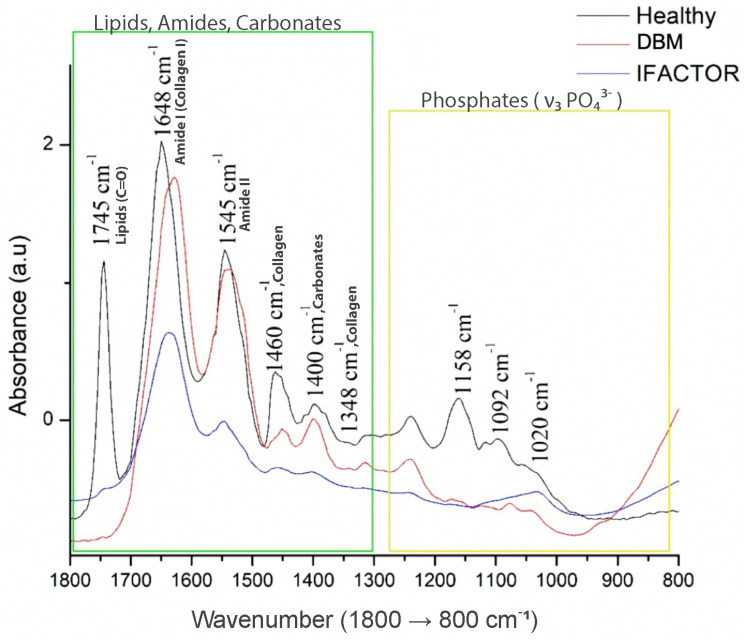
FTIR spectra comparing healthy bone (Healthy) with experimental groups (DBM and iFactor^®^) in the fingerprint region (1800–800 cm^−1^). Key absorption bands identify biomolecular differences: (1) The stronger lipid (1745 cm^−1^) and collagen type I (1648 cm^−1^) signals in healthy bone indicate better preservation of organic matrix components. (2) DBM shows intermediate collagen content (1648, 1545 cm^−1^) closer to healthy bone than iFactor^®^, suggesting superior matrix preservation. (3) Reduced phosphate bands (1158–1020 cm^−1^) in both experimental groups reveal deficient mineralization, though carbonate substitution (1400 cm^−1^) was higher in DBM, indicating distinct mineral modification patterns.

The averages of the raw and normalized FTIR spectra of the healthy bone group and the experimental groups (DBM and iFactor^®^) are shown in Figure 5, where the fingerprint region (1800–800 cm^−1^) is depicted. Bands associated with bone biomolecules were identified, such as lipids, proteins (collagen type I), and minerals (phosphates and carbonates). In the region from 1800 to 1300 cm^−1^ (remarked in green), lipids, amide groups related to collagen, and carbonates were evidenced. At 1745 cm^−1^, the absorption bands related to the extension vibrations of the C=O ester group of lipids showed a higher absorbance in the healthy group than in the experimental groups; in the same way, the peak at 1648 cm^−1^, associated specifically to collagen type I, showed a higher absorbance in the healthy group than in the experimental groups, with the DBM group showing a higher amount than the iFactor^®^ group; this was also observed in the band at 1545 cm^−1^, which is related to Amide II, and the bands at 1460 and 1348 cm^−1^ associated with collagen, and also in the band at 1400 cm^−1^ related to carbonate. In the same way, the range from 1200 to 800 cm^−1^ corresponds to the phosphate groups on the bands at 1158, 1092, and 1020 (marked in yellow); all these peaks exhibited a higher absorbance in the HB group compared to the experimental groups. Therefore, considering all the biomolecular bone compounds, we found that the biomolecular components of the DBM group were more similar to the healthy bone group (see Figure 5).

### 3.3. Comparative Analysis of FTIR Results

**Figure 6:**
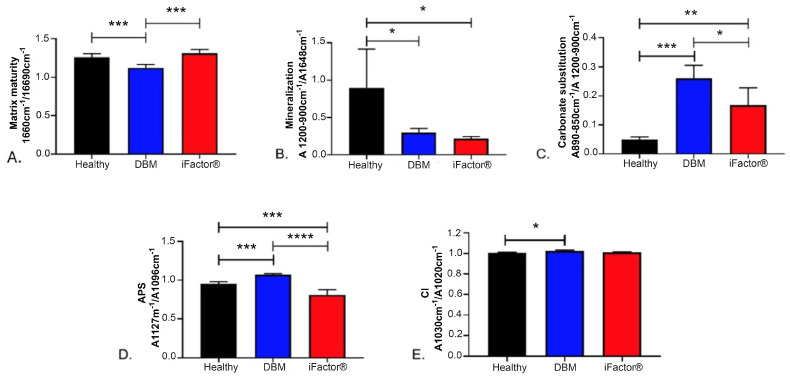
Comparative FTIR analysis of (**A**) matrix maturity, (**B**) mineralization, (**C**) carbonate substitution, (**D**) acid phosphate replacement (APS), and (**E**) crystallinity index. Key findings: (1) DBM’s significantly lower collagen cross-linking (**A**, *p* < 0.0005) and higher carbonate substitution (**C**, *p* < 0.05) suggest altered matrix remodeling compared to healthy bone. (2) Both groups showed impaired mineralization (**B**, *p* < 0.05), but DBM’s higher crystallinity index (**E**, *p* < 0.05) indicates more organized mineral crystals. (3) The divergent APS values (**D**) highlight fundamental differences in phosphate chemistry—DBM’s increase (*p* < 0.0005) may reflect residual processing effects, while iFactor^®^’s decrease (*p* < 0.00005) suggests distinct mineral properties. A *p*-value < 0.05 was used to establish that there is a statistically significant difference. * = *p* < 0.05, ** = *p* < 0.005, *** = *p* < 0.0005, **** = *p* < 0.00005.

After performing the analysis of the results obtained by FTIR, we observe that when comparing the matrix maturity, the mature-to-immature collagen cross-link ratio was lower in the DBM group compared to the healthy bone group (*p* < 0.0005), with no statistical significant difference in the iFactor^®^ group compared to the healthy bone group (Figure 6A). Regarding the mineralization, the mineral-to-matrix ratio in the two experimental groups significantly decreased compared to the healthy bone group (*p* < 0.05) (Figure 6B). For the carbonate substitution, the carbonate-to-phosphate ratio showed an increase in the experimental groups, with the DBM group exhibiting a greater amount than the iFactor^®^ group (*p* < 0.05) (Figure 6C).

In relation to the absorbance values observed in the acid phosphate replacement (APS), the DBM group showed a greater amount than the healthy bone group, while the iFactor^®^ group showed a lower amount than the healthy bone group (*p* < 0.0005 and *p* < 0.00005, respectively) (Figure 6D). Finally, the crystallinity index of the analyzed sample (CI) for the DBM group showed a significant increase compared to the healthy group (*p* < 0.05) (Figure 6E).

### 3.4. Main Trends Established Through FTIR Spectra Analysis

Through the analysis of the results obtained in the experimental groups, the main trends in the FTIR spectrum could be identified, which showed that the healthy bone group exhibited the highest absorbance across most biomarkers, reflecting its well-preserved biochemical profile. Notably, lipids (1745 cm^−1^) and collagen type I (1648 cm^−1^) showed significantly stronger signals in healthy bone, suggesting robust structural integrity. In contrast, both DBM and iFactor^®^ groups displayed reduced absorbance for these components, with DBM demonstrating intermediate collagen content closer to healthy bone, while iFactor^®^ showed the lowest collagen signal. On the other hand, carbonate (1400 cm^−1^) and phosphate (1158–1020 cm^−1^) bands further highlighted differences in mineral composition. Healthy bone had the highest carbonate and phosphate absorbance, indicating optimal mineralization. The experimental groups showed reduced phosphate levels, confirming lower mineral content. Interestingly, carbonate-to-phosphate ratios were elevated in DBM and iFactor^®^, suggesting increased carbonate substitution (a common feature in less mature or remodeled bone). Taking into account the trends found through the FTIR analysis, it indicates that DBM mimicked healthy bone in collagen content and carbonate substitution more closely, while iFactor^®^ showed greater deviations in biomolecular composition. These findings highlight DBM’s potential as a biomimetic material, although neither experimental group fully replicated the biochemical profile of native bone.

## 4. Discussion

According to our results, the formation of calcium nodules (an indicator of osteogenic differentiation) was observed in both experimental groups (DBM vs. iFactor^®^). These results suggest that both scaffolds support osteogenesis, albeit with different degrees in the speed of bone regeneration, since accelerated bone growth has been observed with P15 through the formation of a greater number of calcium nodule aggregates identified in the extracellular matrix of mesenchymal stem cell cultures exposed to iFactor^®^ compared to cultures enriched with DBM. This could be mainly caused by adhesion, cell proliferation, and the activation of integrin receptors promoted by the P15 peptide.

Bhatnagar et al. (1999) [17] demonstrated that the incorporation of P-15 into an anorganic bone matrix significantly improves cell adhesion and proliferation. Cells cultured on P-15-coated matrices showed organized three-dimensional colony formation and early mineralization using Alizarin Red staining. This finding confirms that P-15 promotes early osteogenesis through enhanced cell adhesion and differentiation, which explains the rapid matrix maturation observed with iFactor^®^. On the other hand, unlike bone maturation, the high mineralization of DBM cultures may be due to calcium residues and the mineral content similar to native bone in the demineralized bone matrix [17].

Implant scaffold compositions are often evaluated as homogenized samples by Fourier transform infrared spectroscopy analysis [25,26], and it has been reported, using FTIR analysis, that the homogenized matrix produced by stem cells (derived from different sources), when plated on various substrates in scaffolds and after osteo-induction, produces a bone-like mineralized matrix [25]. The analysis revealed important differences in the biomolecular composition of the scaffolds compared to healthy bone. Both iFactor^®^ and DBM exhibited lower mineral-to-matrix ratios, indicating reduced mineralization compared to native bone. However, DBM demonstrated biomolecular properties that were more similar to healthy bone, particularly in terms of collagen content and carbonate substitution.

Both experimental scaffolds exhibited lower matrix maturity compared to healthy bone, with DBM showing a marginally closer resemblance to native bone. However, this comparison omits the rapid maturation of bone matrix as a key parameter for the clinical success of grafts. This could be supported by the matrix formed by observing well-organized cell colonies and a highly oriented matrix in cells cultured on a P-15-coated substrate. These findings reinforce the fact that iFactor^®^ offers a substantial clinical advantage by providing faster mechanical fixation and greater initial stability.

Matrix maturity, determined by the collagen cross-link ratio, is critical for the mechanical strength and biological function of bone. While neither scaffold achieved the maturity levels of native bone, the slight advantage of DBM may enhance its biomechanical performance in vivo.

The lower mineralization observed in both scaffolds could be a limitation for applications requiring immediate load-bearing capacity. However, the increased carbonate substitution in DBM suggests a potential for improved bioactivity and remodeling over time, as carbonate incorporation into the bone matrix is known to influence osteoclast-mediated resorption and new bone formation.

The in vitro nature of the experiments may not fully capture the complex interactions that occur in vivo. Additionally, the use of a single cell type and the relatively short culture duration limit the generalizability of the findings. Future studies should include in vivo models to assess the clinical efficacy of these scaffolds, considering factors such as vascularization, immune response, and long-term remodeling. Local antibiotic delivery with demineralized bone matrix studies demonstrates that this osteoinductive and biodegradable material can be loaded with gentamicin and release clinically relevant levels of the drug for at least 13 days in vitro. It has also been described that adding an antibiotic mixed with the DBM does not affect its osteoinductive potential, either in vitro or in vivo [27].

## 5. Conclusions

iFactor^®^ and DBM demonstrated similar osteogenic potential after 3 months of follow-up. Although DBM has a closer biomolecular composition and carbonate substitution compared to healthy bone, iFactor^®^ showed faster matrix maturity expressed through the formation of a greater number of calcium nodule aggregates and higher hMSCs proliferation.

At the time of our study, the available literature on iFactor^®^ (combined with other therapeutic components such as antibiotics or growth factors such as BMPs) compared with DBM was lacking. Thus, healed infected bone rates in fractures within research on the comparison of biocompatible carriers will be attractive for the development of clinical trials.

## Data Availability

Data are available from the corresponding author upon reasonable request.

## Abbreviations

The following abbreviations are used in this manuscript:

DBM	demineralized bone matrix
FTIR	Fourier transform infrared spectroscopy
hMSCs	human mesenchymal stem cells
AATB	American Association of Tissue Banks
ABM	anorganic bone mineral
APS	acid phosphate replacement
CI	crystallinity index
HU	Hounsfield unit
